# Study on the Anticancer Activity of Prodigiosin from Variants of *Serratia Marcescens* QBN VTCC 910026

**DOI:** 10.1155/2022/4053074

**Published:** 2022-04-25

**Authors:** Sy Le Thanh Nguyen, Tien Cuong Nguyen, Thi Tuyen Do, Trong Luong Vu, Thi Thao Nguyen, Thi Thao Do, Thi Hien Trang Nguyen, Thanh Hoang Le, Dinh Kha Trinh, Thi Anh Tuyet Nguyen

**Affiliations:** ^1^Institute of Biotechnology, Vietnam Academy of Science and Technology, 18 Hoang Quoc Viet Road, Caugiay District 10600 Hanoi, Vietnam; ^2^Thai Nguyen University, Vietnam; ^3^Thuyloi University, Vietnam

## Abstract

Prodigiosin (Pg), a secondary metabolism produced by numerous bacterial species, is known as anticancer, antibacterial, antifungal, immunosuppressant, antioxidant, antimalarial properties. Pg has been tested for antitumor activity in many different cancer cell lines but studies in LU-1, KB cell lines, and tumor-bearing mice are still limited. In this study, *Serratia marcescens* QBN VTCC 910026 strain (GenBank: KX674054.1) was mutated using Ethyl Methanesulfonate (EMS) to increase the production of Pg. One strain known as EMS 5 was capable of increasing prodigiosin biosynthetic yield by 52% when compared to the wild-type strain. Red bacterial pigmented colonies containing Pg were collected from solid media, lysed with acetone, purified with toluene: ethyl acetate at a ratio of 9: 1 (v/v), and then used to evaluate the potential anticancer activity. The purity of Pg was confirmed using a high-performance liquid chromatography (HPLC) method which indicated a 98% rate. Pg chemical formula which was determined using ^1^H-NMR and ^13^C-NMR spectroscopy, confirmed as prodigiosin (Pg). Human breast cancer cell lines MCF-7, oropharyngeal cancer KB, and particularly lung cancer LU-1 in vitro were used to test the anticancer activity of purified Pg compound. It showed a strong inhibitory ability in all the cancer cell lines. Furthermore, the isolated Pg had capable of inhibiting tumor growth, the tumor volume decreased by 36.82%, after 28 days. The results indicated that the bacterial prodigiosin from variants *Serratia marcescens* QBN VTCC 910026 strain is an encouraging fragment suitable for therapeutic applications.

## 1. Introduction

Prodigiosin (Pg) is a natural secondary metabolite that is biosynthesized by Gram-negative and Gram-positive bacteria such as *S. marcescens*, *V. psychroerythrus* [1], *V. ruber* sp. *nov, V. gazogenes* ATCC 29988 T and some other species such as *Streptomyces coelicolor* [2], *Pseudoalteromonas* sp. 1020R [3], *Janthinobacterium lividum* [4], *Zooshikella rubidus* S1-1 [5]. Prodigiosin is a tripyrrole rings pigment forming a pyrryldipyrrylmethane skeleton (two of the rings are directly linked to each other and the third is attached by way of a methane bridge) synthesized by *S. marcescens* with the molecular formula is C_20_H_25_N_3_O and a molecular weight of 323.44 Da. It is sensitive to light and insoluble in water. It is moderately soluble in alcohol and ether, and soluble in chloroform, methanol, acetonitrile and DMSO Three compounds, namely prodigiosin, undecyl prodigiosin and cycloprodigiosin hydrochloride, form the prodigiosin group and have biological activities for immunosuppression and apotosis induction of cancer cells. Cytotoxic effect of them requires the C-6 methoxy substituent. The A-pyrrole ring plays an important role both in co-nuclease activity and cytotoxicity of prodigiosin [6]. Pg and Obata x (OBX), two members of their prodiginine, small molecules active against cancer clinical trials are studying [7]. Pg has been receiving much attention from researchers due to its immunosuppressive and anticancer properties on many drug-resistant cancer cell lines such as MDR1, BCRP, or MRP2 [8], or K562 human chronic myelogenous leukemia cells [9]. In addition, Pg has antibacterial and antifungal activities [10] and has little effect on normal cells. Pg has been tested for antitumor activity in more than 60 different cancer cell lines and the average IC_50_ value was 2.1 *μ*g/mL. The anti-cancer potential of Pg was assumed to be due to this active substance causing apoptosis in cancer cells. Pg was found to be present in small granules near the cell nucleus, in the nucleus [11], cytoplasm [12], and the mitochondrial membrane [13], Pg has been shown to induce apoptosis in cells by four pathways: DNA damage, cell pH changes, cell cycle changes, and signal transduction interference. Pg intercalates into the DNA sequence (prefers AT sequence) in the small groove position, along with the presence of Cu^2+^ will promote oxidation leading to DNA damage and leading to cell death [14]. The bacterial prodigiosin induces apoptosis in JEG3 cells in vitro. and significantly inhibited the growth of JEG3 and PC3 cells in vivo experiment. The expressions level of protein makers such as P53 and Bax/Bcl-2 in JEG3 and PC3 were significantly higher than in untreated groups [14]. In this paper, we generated the variants of *Serratia marcescens* strain which was isolated from soil in Viet Nam. After purification and identification of Pg this compound was tested the anticancer activities in vitro and in vivo. Our results indicated that the Pg from variants of *S. marcescens* isolated from solid in Viet Nam possessed strong activities against cancer cells lines, tumor with high promising as an anticancer drug in future.

## 2. Materials and Methods

### 2.1. Chemical Reagents

Chemical, column chromatography Silica gel and solvents (methanol, ethyl acetate, chloroform, acetone, toluene) used in this study were from Merck (Darmstadt, Germany) or other suppliers (China). Thin-layer chromatography was performed on silica gel plates with 0.25 mm thick silica gel 60 F254 (Merck). Standard prodigiosin was provided by Sigma Aldrich (St. Louis, MO, USA). The strain of *Serratia marcescens* strain QBN VTCC 910026 (GenBank: KX674054.1) was isolated and identified by the members of the Enzyme Biotechnology laboratory, Institute of Biotechnology (Vietnam Academy of Science and Technology - VAST) in previous studies.

### 2.2. *S. Marcescens QBN VTCC 910026* Culture

*S. marcescens* stored in glycerol 25% at -84°C was transferred into a cooler tray at -20°C. The bacteria were then streaked onto a LB agar dish for activation, followed by incubating at 28°C for 24 h until red colonies appeared. Next, a separated colony was isolated for shake culture in LB at 200 rpm, 28°C for 16 h. After that, *S. marcescens* was spread onto petri dishes of peanut medium using a glass spreader. The dishes were then incubated for 3 days until red pigment can be visibly seen. For Pg production, variant *S. marcescens* was grown on 2% peanut seed powder and 2% agar, then incubated on the tray (20 x 30 cm) at 28°C for 48 h. Cells grown on the surface was collected for Pg extraction and purification.

### 2.3. EMS Mutagenesis

*S. marcescens* strain QBN VTCC-910026 was subjected to chemical mutagenesis using ethyl methyl sulfonate (EMS) [15, 16]. Mutagenesis was achieved by inoculating LB medium with fresh culture of the selected isolate of *S. marcescens* and incubated at 28°C for 24 h. Then cell supernatants were centrifuged at 4000 rpm in 20 min at 4°C to obtain the pellets. These pellets were mixed with EMS at serial concentrations of 400, 800, 1600, 3200, or 6400 *μ*g/mL overnight at 37°C. After incubating, the pellets were obtained by centrifugation at 12500 rpm for 10 min at 4°C. Washing these pellets with 0.2% Tween 20 from 4 to 6 times. Washed pellets were dissolved into 200 *μ*L 0.2% Tween 20.50 *μ*L of cell supernatants were used to spread evenly over the surface of the agar in each dish. Cultured dishes were inoculated at 28°C for 24 h.

### 2.4. Cell Lines and Cell Cultures

Metastatic Lewis lung carcinoma (LLC) cell line was used to induce cancer in mice (provided by Dr. Jeanette Maier, University of Milan, Italy). Fibroblast, HepG2, H460, LU-1, KB, and MCF-7 is supplied from American Type Culture Collection (ATCC) and grown at 37°C in the humidified atmosphere with 5% CO_2_. RPMI (Gibco, USA) supplementing with 10% fetal bovine serum (FBS) and 1% antibiotics was used to culture cancer cell lines. For prodigiosin treatment, cells were cultured until the confluency reached 80% and washed with PBS before carrying out the experiments.

### 2.5. Extraction, Purification and Identification of Prodigiosin

10 g of the wet cell was collected from a peanut-agar medium. The ethyl acetate: acetone (1: 1) solvent was selected to extract the red pigment from the cell of *S. marcescens* EMS 5 with the ratio of 1: 20 (w/v). The mixture was kept on a shaker at 200 rpm for 3 h at 28°C, and then centrifuged at 4000 rpm at 4°C for 15 min. After the sample was extracted 6 times, 300 mg of dry sediment was collected for purification with column chromatography (CC). In this study, the crude extract was mixed with SiO_2_ at a ratio of 1: 1 (w/w), then was put on a column pre-immersed with absolute methanol. The extract in the column was eluted by toluene: ethyl acetate solvent system at a ratio of 9: 1 (v/v). Each segment passed through the column was collected with a volume of 50 ml, then checked for the presence of prodigiosin by thin layer chromatography. The identified segments were collected in groups to continue passing through the column or to dry for further studies. Thin layer chromatography was performed on a silica gel plate with 0.25 mm thick silica gel 60 F254. The solvent system was n-hexane: ethyl acetate (1: 1) as the mobile phase. The compounds were visible by iodine staining. Prodigiosin purity was determined by HPLC. 10 *μ*l of sample was applied to the system. The purification experimental design was determined by LC/MS 1100 Agilent Ion sources ESL, column ODS C18, 3.0 x 150 mm, 3.5 *μ*m, mobile phase MeOH: H_2_O (80: 20; v/v). NMR 1D and 2D spectra were measure by Bruker AV 500 MHz in CDCl_3_ solvent. ^1^H NMR and ^13^C NMR were determined at 500 MHz and 125 MHz, respectively. Tetramethylsilane (TMS) was used as the internal standard. The signals were obtained as singlet (s), doublet (d), double doublet (dd), triplet (t), quintet (quint), and multiplet (m). MS spectra was measured by LC-MS Agilent 1100 (USA). The culture broth was analyzed by HPLC mobile phase in MeOH: H_2_O =20: 80 for 2 min, MeOH: H_2_O =20 – 100/80 – 0 for 17 min, MeOH: H_2_O =100: 0 for 8 min, MeOH: H_2_O =20: 80 for 5 min. The quantity of Pg in the culture broth was determined on HPLC based on standard Pg. Standard prodigiosin (Sigma) was diluted at various concentrations. Then, use a spectrophotometer to measure the absorbance at 535 nm.

### 2.6. Proliferation Assay

Concentration dependent effects on cell proliferation were measured using the MTT (3-(4,5-dimethylthiazol-2-yl)-2,5-diphenyl tetrazolium bromide) assay. Cancer cells after culture were transferred to 96 wells plate with a density of 2 x 10^4^ cells/well and grown for 24 h at 37°C. Then, the culture media of cancer cells were replaced by a fresh one supplementing with different concentration of prodigiosin of (0.5; 1; 2; 4; 6; 8 and 10 *μ*g/mL) for another 24 h. The control was used without adding prodigiosin. At the end of treatment, 5 mg/mL of MTT reagent was added to each well and incubated for 3 h. The MTT containing supernatant solution was removed and 100 *μ*L of DMSO was added to each well to dissolve violet formazan crystal. The proliferation assay was measured at OD_595_. Based on MTT results, we calculated the inhibitory concentration by using different concentrations of prodigiosin and the control without prodigiosin. The software TableCurve2Dv4 was used to determine IC_50_ value. Cell viability rate = (Δ_sample_ − Δ_media_/Δ_control_ − Δ_media_) × 100(%)

Δ_*sample*_: Absorbance value of cancer cells treated with prodigiosin measured at OD_595_.

Δ_*control*_: Absorbance value of cancer cells without treated with prodigiosin measured at OD_595_.

Δ_*media*_: Absorbance value of media measured at OD_595_.

### 2.7. Tumor Suppression Capacity of Pg Using Tumorized Mice Model

Tumor induction of mice with the LLC cell line.

LLC cells were grown in DMEM medium supplemented with 10% bovine serum and 1% antibiotics at 37°C and 5% CO_2_. Cells were harvested and injected into the thighs of mice at a concentration of 2x10^6^ cells/mice (which is the tumor-causing concentration for mice reaching 100%). After 5 days of injecting LLC cells, if there is a tumor, then mice were used for next experiment. 18 healthy BALB/c mice, aged 10-12 weeks old, injected with LLC cells above were weighed, measured for tumor size at the injection site of LLC cells, and then divided to 3 groups (6 mice/group) [17]. After 5 days of tumor induction, mice were randomly divided into three groups which were (i) pathological control group receiving water with a volume of 0.3 mL/mouse; (ii) reference group receiving capecitabine at 200 mg/kg body weight (bw.) orally; (iii) sample treatment group receiving of Pg at 1 mg/kg bw. Tumor suppressive capacity of tested samples were compared with the pathological control group. The experimental mice were weighed and measured primary tumor sizes at the injection site every 7 days. The tumor volumes were calculated using the formula of previous studies [14, 15] as:V = a × (b^2^/2)

(V: tumor volume; a: length of the tumor; b: diameter of tumor)

## 3. Results

### 3.1. Mutagenesis on *Serratia Marcescens* QBN VTCC 910026

Ethyl methanesulfonate (EMS) was used as a mutagenic agent to produce a hyper producing strain in chemical mutagenesis experiments. *S. marcescens* strain QBN VTCC-910026 (GenBank: KX674054.1) was treated by EMS (Ethyl methane sulfonate) at different concentrations to generate variants lines. Some of variants produced Pg more than the wild type. However, most differences were not significant meaningful in statistic. The only one strain that produce Pg more than the wild type strain 52% (858 mg/L) was *S. marcescens* EMS 5 (Supplement 1). The prodigiosin productivity about 1.52 fold for the mutant *S. marcescens* EMS 5 compared with productivity of the wild type.

### 3.2. Purification and Identification of Prodigiosin from *S. Marcescens* EMS 5

**Figure 1:**
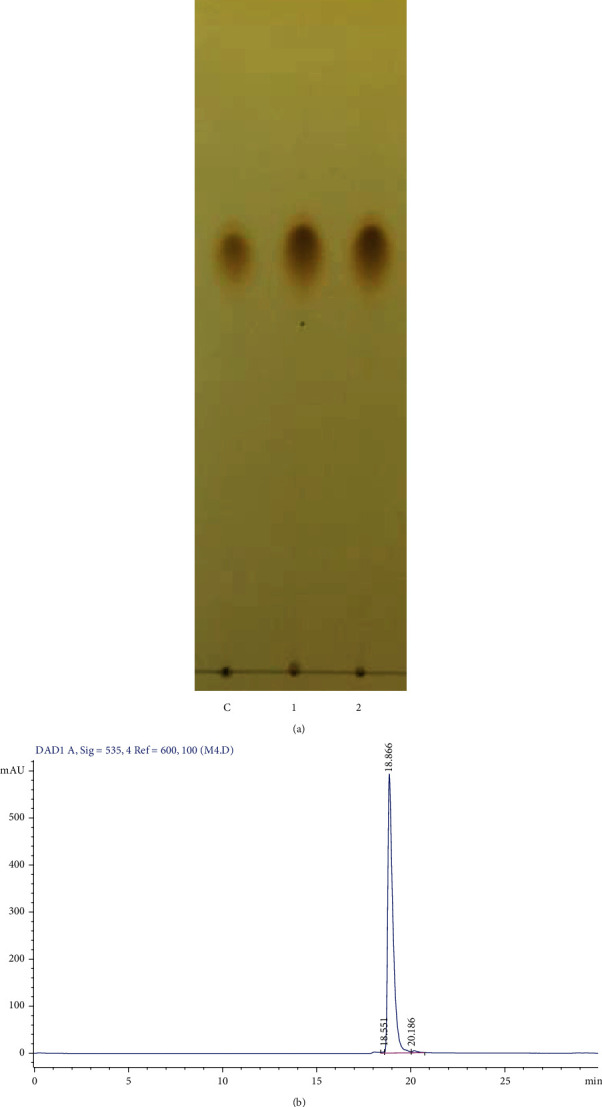
Purification of Prodigiosin. (a) TLC chromatography of the purified prodigiosin passing through the silica-gel column; lane 1: standard Pg, lane 2, 3: purified Pg. (b) HPLC of purified prodigiosin.

An effective solvent system for Pg extraction from the culture of *S. marcescens* M10 was determine [18]. In this study, we have used the solvent ethyl acetate: acetone (1 : 1) to extract the prodigiosin from *S. marcescens* EMS 5. The cell-free extract was loaded on the silica gel column. 9 fractions were collected and checked on a TLC chromatography plate. The fractions 6–9 restrained a visible band corresponding to the standard prodigiosin as control (Supplement 2). The prodigiosin-contained fractions were collected and purified by passing the new silica gel column a second time. Two fractions were collected, and one single band of the putative compound was shown (Figure 1(a)). The HPLC result indicated that the purified compound harbored 1 single peak of pure prodigiosin reached 98% (Figure 1(b)).

The compound was isolated in the form of purple-red powder, melting temperature of 151-152°C. The spectral data indicated a peak of m/z [M + H]^+324.1^ (m/z) corresponding to a molecular formula C_20_H_25_N_3_O (Supplement 3).

**Figure 2:**
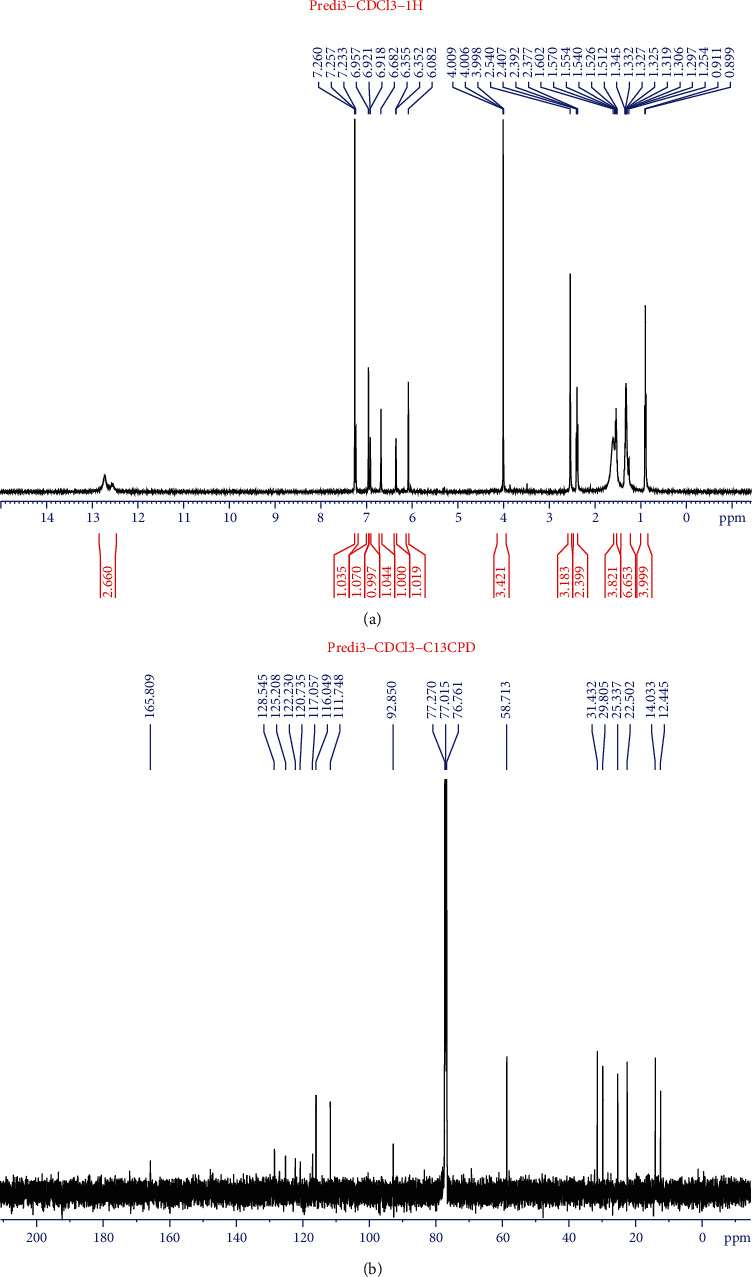
Spectra of purified compound. (a). ^1^H NMR proton spectrum. (b). ^13^C NMR spectrum of active prodigiosin purified from *S. marcescens* EMS 5.

The ^1^H NMR spectral data of purified compound from (Figure 2(a)) *S. mercescens* EMS 5 harboring broad singlet signals of 2 hydrogen atoms bind a nitrogen atom at 12.57 ppm (^1^H) and 12.70 ppm, which corresponding to H-1and H-1. In the region of aromatic hydrogen and olefin, there are 6 resonant signals corresponding to 6 protons. Among of them, there are 3 singlet signals at *δ*_H_ 6.96, 6.68, and 6.08 ppm. The other 3 signals correspond to 3 protons belonging to the same spin system. The COSY ^1^H-^1^H spectra (Supplement 4) indicated the proton belonging to A ring: *δ*_H_ 7.23 (H-5), 6.92 (H-3) and 6.35 (H-4). The interaction constants of these protons were small enough (1.5 Hz), characterizing a heterozygous 5^th^ ring, such as a pyrrole one.

In the high field, there are signals of an OMe group (s, 4.00), a Me group at *δ*_H_ 2.5 (s), a group of Me terminal carbon (t, 0.90), 2 groups of CH_2_ (t, 2.39 and 1.54), and 2 groups of CH_2_ in the form of overlay multiple at *δ*_H_ 1.32. The COSY spectra allow the identification of a spin system of an n-pentyl group.

Besides, there was an interaction of methyl group appeared on the COSY spectra (*δ*_H_ 2.54) and a CH_2_ group (*δ*_H_ 2.39) with proton *δ*_H_ 6.68. This allowed us to define H-4” (*δ*_H_ 6.68) and CH_3_-6”, CH_2_-7” which belonging to the C ring.

The ^13^C NMR (Figure 2(b)) and HSQC (Supplement 5) showed resonance signals of 20 carbon including 13 carbon sp^2^ (6 CH and 7 quaternary carbons) and 1 methoxy group (*δ*_C_ 58.7), 2 methyl groups and 4 CH_2_ groups. The HSQC spectra allowed the determination of the corresponding hydrogen-bound carbon. Specifically, in the A ring: 6.92/117.1 (H/C-3), 6.35/111.7 (H/C-4), 7,23/125,9 (H/C-5), OMe groups in the B ring at 6.08/92.9 (H/C-3'), 4.00/58.7 (H/C-7'), a CH olefin group at 6.96/117.1 (H/C-6'). In the C ring there is a CH group at 6.68/128.4 (H/C-4”), a methyl group 2.54/12.4 (H/C-6”), and corresponding groups of pentyl circuits.

The HMBC spectra (Supplement 6) determined the quaternary C (C-2, 120.7) of an A ring from the interaction of H_4_-C_2_, H_5_-C_2_, quaternary C of B ring (C2', 146.4; C-4', 165.8; C-5', 120.7) by the interaction of H-3'-C-2', C-5', H-6'-C-4', quaternary of C ring (C-2”, 145.3; C-3”, 125.2; C-5”, 128.5) by the interaction of H-4”-C-2” and C-3”, H-6”-C-2” and C-4”. The proton interactions at 6.96 with C-4' and C'4” defined H-6'.

**Figure 3:**
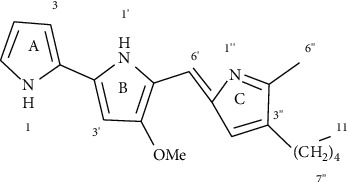
Chemical structure of purified prodigiosin.

A methyl group of H-6” and a methylene group of H-7” had an interaction in HMBC with C-2”and C-4”. From the above MS and NMR spectra, we can conclude the purified compound was Pg (Figure 3).

### 3.3. Inhibitory Activity of Pg against Cancer Cell Lines

With IC_50_ values on fibroblast normal cell lines more than 20 *μ*g/ml (equivalent to 61.84 *μ*M), purified Pg from *S. marcescens* EMS 5 had no effect on normal cells. Therefore, it's very potential to become medicine for cancer treatment.

**Figure 4:**
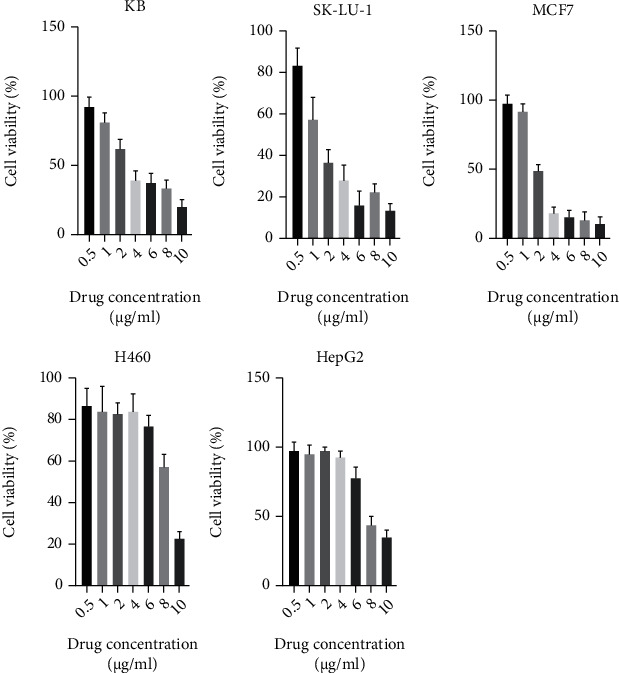
Inhibitory concentration of Prodigiosin (0.5; 1; 2; 4; 6; 8 and 10 *μ*g/ml) on different cancer cell line KB, SK-LU-1, MCF7, H460 and HEPG2.

The IC_50_ value of Pg for HepG2 cancer cells, which was 8.75 *μ*g/ml, equivalent to 27 *μ*M, showed that purified Pg from *S. marcescens* EMS 5 strain did not have cytotoxic activity and had a weak inhibitory effect on the growth of HepG2 cancer cells (IC_50_>4 *μ*g/mL). Prodigiosin was resistant to H460 cells at the concentrations from 6 *μ*g/mL to 10 *μ*g/mL. The IC_50_ value of purified Pg reached 7.7 *μ*g/mL, equivalent to 23 *μ*M. The purified Pg was strongly resistant to MCF-7 cells at a concentration of 4-10 *μ*g/mL. At the concentration of 2 *μ*g/mL, purified Pg inhibited more than 50% of cells (IC_50_<2 *μ*g/mL). For SK-LU-1 cells, IC_50_ was 1.5 *μ*g/mL, equivalent to 4.6 *μ*M. Thus, purified Pg had cytotoxic activity and could inhibit the growth or killing of SK-LU-1 cancer cells. In KB carcinoma cells, at a concentration of 4 *μ*g/mL, all cells shrank, and changed shape and some cells died. The cell viability treated with Pg at 4 *μ*g/mL reached 40%. However, at the concentration of 10 *μ*g/mL, this number accounted for 20% (Figure 4).

### 3.4. Tumor Growth in Experimental Groups

**Figure 5:**
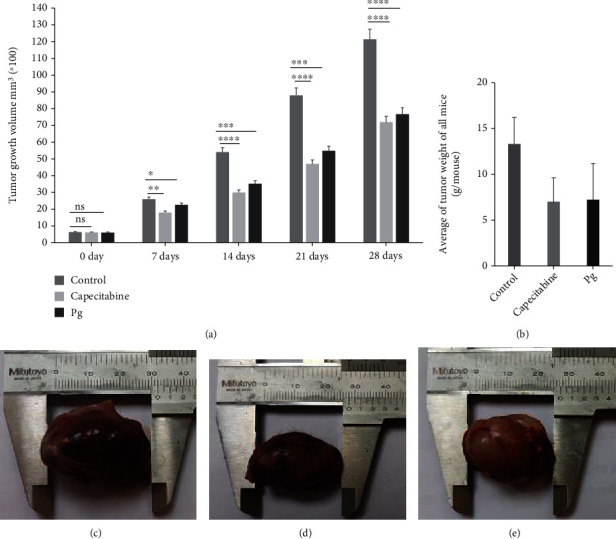
The effect of Pg on tumor in the thigh of mice before and after treatment. (a). The effect of Pg on tumor growth of mice after 28 days. Control: Healthy mice. Capecitabine: Mice were orally administrated with capecitabine at the dose of 200 mg/kg/day. PG: Mice were received with Pg at the dose of 2 mg/kg/day. Significance was determined using an unpaired two tailed t test: ∗∗∗∗p <0.0001, ∗∗∗p <0.001, ∗∗p <0.005, ∗p <0.05. Ns: Non-significant. Each value is expressed as mean ± SD. n = 10 per group. The average of tumor weight of all mice. (b). Tumor from mice without drug treatment. (c). Tumor was from mice orally administrated with capecitabine at the dose of 200 mg/kg. (d). Tumor was from mice injecting with Pg at the dose of 1 mg/kg (e).

Tumor growth is a crucial indicator to assess the antitumor effect of the reagent. The results on the tumor in the thigh of mice are shown in Figure 5. The results (Figure 5 a) indicated that tumor volumes of the group administrated with Pg at the dose of 1 mg/kg/day, one time per 2 days: the 7^th^ reduced compared to the control group. However, no statistical difference was found (*p* >0.05). The same result was observed when comparing these two groups at the 14^th^, 21^st^ and 28^th^ day, but the difference was statistically significant (*p* <0.05). The tumor volume of mice in the reference group orally treated with capecitabine at the dose of 200 mg/kg/day decreased at all measured points on the 7^th^, 14^th^, 21^st^, and 28^th^. The statistical difference of the tumor volume in this group was found (*p* <0.05). The tumor volume of mice in the reference group that used capecitabine at the dose of 200 mg/kg/day remarkably decreased compared to the control group on the 7^th^, 14^th^, 21^st^, and 28^th^. The statistical difference in this group was found (*p* <0.05). Again, as shown in Figure 5, the average tumor weight of all mice administered with Pg decreased to 7.23 g/mouse, compared with 13.31 g/mouse of the control group.

## 4. Discussions

The red pigment Pg is of great interest to many scientists and research because of its great potential in medicine. Pg has immunosuppressive, anticancer, antibacterial, antifungal, and antioxidant activity [14, 19]. This study reported that variants of *Serratia marcescens* QBN VTCC 910026 strain produce much more Pg production of 858 mg/L compared to HDZK-BYSB107 (0.656 g/L), *Zooshikella rubidus* S1-1 (0.048 g/L) and *Hahella chejuensis* KCTC 2396 (0.028 g/L) [5, 14, 20]. The Pg in *S. marcescens* were packed inside 2 membranes of peptidoglycan and lipopolysaccharide [11] so a solvent mixture of EtOAc and HCl 1% to extract the intracellular substances was used [12]. However, this solvent was not suitable to extract Pg from *S. marcescens* EMS 5. Consequently. a solvent system of ethyl acetate: acetone at a ratio of 1 : 1 (v/v) were used to disturb bacterial cell membrane and extracted Pg from *S. marcescens* EMS 5. The suitable solvent system was run on TLC chromatography was chloroform: ethyl acetate at the ratio of 1: 1 to separate compounds in crude pigment. This result is similar to Park's announcement in 2012 on choosing an appropriate solvent to extract Pg from cells of strain *H. chejuensis* M3349 [21]. Previously, Pg was pre-extracted in the chloroform phase and eluted by toluene and ethyl acetate, the purified compound was obtained by HPLC reached 14.3 g/L. One advantage point of this study is that the culture of *S. marcescens* was carried out in an internal adsorbent bioreactor built by the research group and the supplement of minerals has boosted the Pg production [22]. Accordingly, fermentation and purification required more steps than our study. Among of the reasons why our Pg production was significantly low because the simple medium and long incubation time took major points for this limitations. In 2012, the Pg production of Park's research team was reduced by half after 2 days of incubation. Besides, Pg production was also affected by other inhibitors in the batch culture [21]. The molecular weight of Pg identified by LC-MS gave a signal peak at 324.1 m/z (Supplement. 2) which is almost similar to Pg from the study of Lin's research team (323.9 m/z) in 2019 [23]. The structure of prodigiosin was established by high-field ^1^H-NMR, ^13^C-NMR spectroscopy. The structure and molecular mass results were similar to prodigiosin from strain HDZK-BYSB107 [14] and prodigiosin from *Serratia* sp. KH-95 [22]. According to the American Cancer Society. The IC_50_ of reagent is below 20 *μ*g/mL (crude extract or chemical fraction) or IC_50_ ≤ 4 *μ*g/mL (pure active substance) is considered as an anticancer agent. Thus, the IC_50_>20 *μ*g/mL (61.84 *μ*M) from *S. marcescens* EMS 5 did not exhibit cytotoxic activity against fibroblast cells isolated from BALB/c mice. Pg has been tested on 60 different cancer cell lines [24], but some cell lines such as LU-1, KB had not been studied. After investigating some HepG2, KB, MCF-7, LU-1, H460 cancer cell lines, we found that purified Pg had a strong inhibitory ability on cancer cell lines: MCF-7 human breast, LU-1 lung cancer, KB carcinoma cells in vitro. The results of our study are also consistent with studies on testing the activity of prodigiosin on cells, which is to change cell shape, cell rupture, and death. The IC_50_ value of the KB line of Pg from *S. marcescens* EMS 5 was equivalent to the activity of Pg from *Labeo rohita* on some other lines. Specifically, Hela was 4.3 *μ*M. HepG2 was 5.2 *μ*M, and KB was 4.8 *μ*M [25]. This value was not much higher than the IC_50_ value of Pg from *S. marcescens* on some other cancer cell lines such as cervical carcinoma (Hela-229) (IC_50_ achieved at 0.7 nM). The anti-tumor activity of prodigiosin compounds has been reported very limited in a few previous studies [14, 18]. The results (Figure 5(a)) showed that our purified Pg significantly reduced tumor volume compared to the control. In our previous research, Pg from *S. marcescens* M10 was injected into the mice abdominal cavity and inhibited 31.18% tumor growth. However, at this dose some mice died on days 19^th^ and 22^nd^. Therefore, in this study, we injected Pg in the mice muscle with lower dose of Pg from *S. marcescens* EMS 5 to 1 mg/kg body weight. The results showed that the inhibitory activity of Pg is not significant difference from our previous study on days 7^th^ and 14^th^. However, on days 21^st^ and 28^th^ the inhibitory activity of Pg from *S. marcescens* EMS 5 was significant inhibit tumor growth (inhibited 36.82% tumor growth after 28 days). The anticancer activity of antitumor compound was reported in other publications. The active ingredient TAT2 extracted from *A. tonkinensis* leaves have been shown to significantly inhibit tumor growth at 100 and 200 mg/kg body weight [26]. Prodigiosin inhibited Wnt/*β*-catenin signaling by targeting various sites in the pathway, including lipoprotein-receptor-related protein (LRP) 6, disheveled (DVL), and glycogen synthetase kinase-3*β* (GSK3*β*). The application of this compound hindered tumor development and decreased the production of phosphorylated LPR6, phosphorylate and unphosphorylated DVL2, Ser9 phosphorylated GSK3*β*, active *β*-catenin, and cyclin D1 [27]. In a combined therapy, prodigiosin/PU-H71 elevated caspases 3; 8; and 9 while decreasing mTOR expression. At the same time HSP90*α*, EFGR, VEGF were downregulated in the expression level. Thus, the compound possessed a high potential for the treatment of TNBC [28]. In an animal model, prodigiosin induced an antitumor activity on human carcinoma (JEG3) and prostate cancer cell line (PC3). The bacterial compound downregulated XIAP, cIAP-1 and IAP-2 [14].

## 5. Conclusions

The red pigment from variants of *Serratia marcescens* QBN VTCC 910026 was extracted and purified from the solid fermentation media by toluene: ethyl acetate solvent system with the ratio of 9: 1 (v/v). The purity of pigment confirmed by the high-performance liquid chromatography (HPLC) method presented as 98%. The red pigment was confirmed as prodigiosin using ^1^H-NMR, ^13^C-NMR spectroscopy. The purified prodigiosin has a strong inhibitory ability on human breast cancer cell lines MCF-7, lung cancer LU-1, oropharyngeal cancer KB in vitro. The prodigiosin from variants of *Serratia marcescens* QBN VTCC 910026 was shown to be capable to decreased the tumor volume by 36.82% compared to the control after 28 days. Thus, we concluded that the prodigiosin of variants of *Serratia marcescens* QBN VTCC 910026 could be deliberated as an anticancer drug in the future.

## Data Availability

The authors confirm that the data supporting the findings of this study are available within the article.

## Supplementary Materials

Supplementary Materials Supplement 1: Prodigiosin concentration; 1: Wild type; 2-36: variants of *S. marcescens.* Supplement 2: TLC chromatography of the supernatant of S. marcescens passing through the first column. S: Cell-free extract of prodigiosin by acetone. 1–9: Fraction 1 to fraction 9 of cell-free extract of prodigiosin when passing through the silica gel column. Supplement 3: MS spectrum of prodigiosin. Supplement 4 COSY spectrum of prodigiosin. Supplement 5: HSQC spectrum of prodigiosin. Supplement 6: HMBC spectrum of prodigiosin.
