# Supplementary material for: Study on the Anticancer Activity of Prodigiosin from Variants of Serratia Marcescens QBN VTCC 910026
Source: Biomed Res Int. 2022 Apr 25;2022:4053074. doi: 10.1155/2022/4053074 (PMC9061010; doi:10.1155/2022/4053074)
Supplement: Supplementary Materials — Supplement 1: Prodigiosin concentration; 1: Wild type; 2-36: variants of S. marcescens. Supplement 2: TLC chromatography of the supernatant of S. marcescens passing through the first column. S: Cell-free extract of prodigiosin by acetone. 1–9: Fraction 1 to fraction 9 of cell-free extract of prodigiosin when passing through the silica gel column. Supplement 3: MS spectrum of prodigiosin. Supplement 4 COSY spectrum of prodigiosin. Supplement 5: HSQC spectrum of prodigiosin. Supplement 6: HMBC spectrum of prodigiosin. [file 4053074.f1.docx]

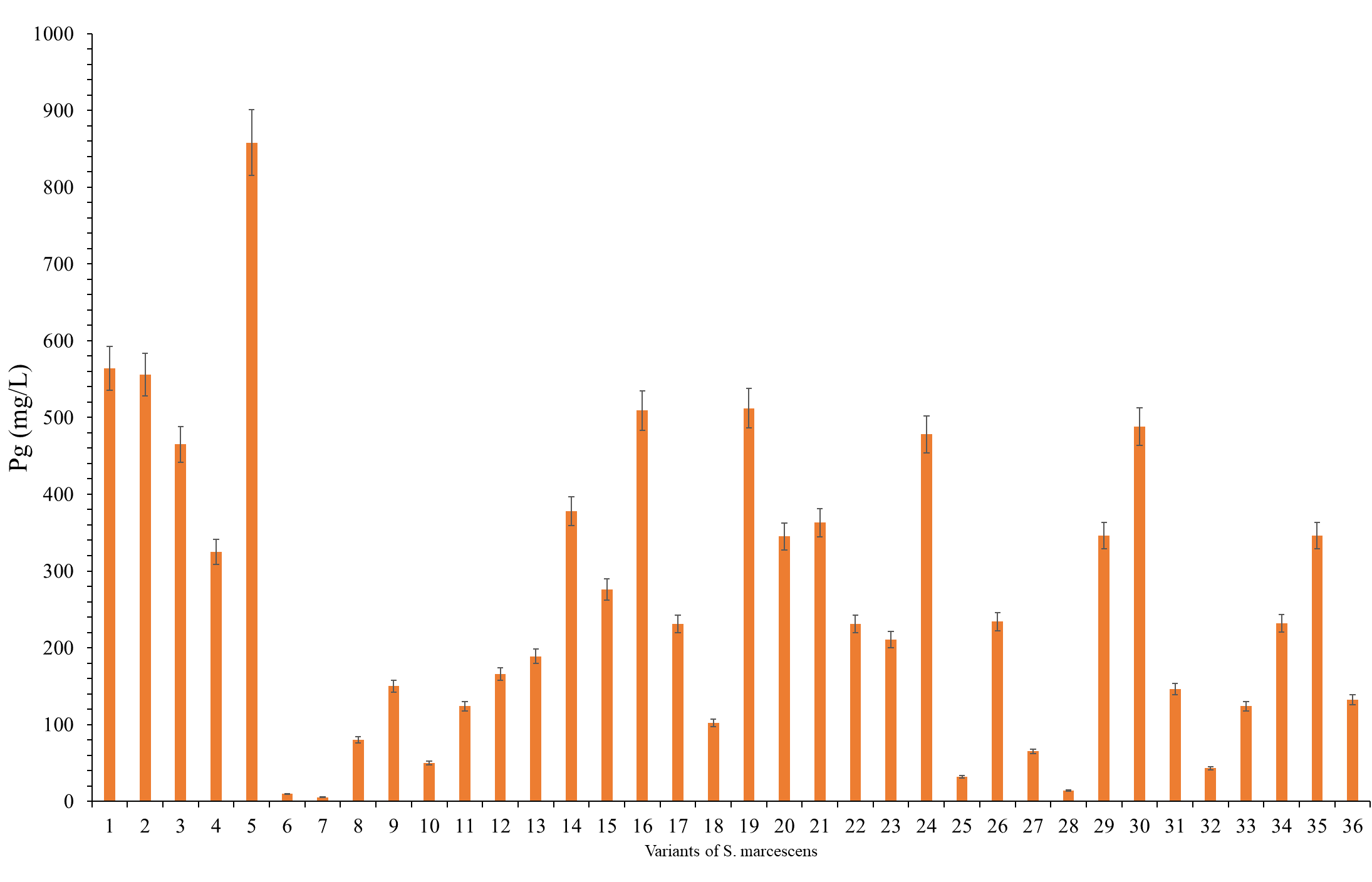


Supplement 1: Prodigiosin concentration; 1: Wild type; 2-36: variants of *S. marcescens*

| 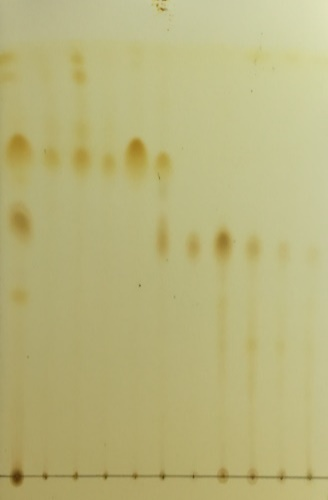 |
| --- |
| S 1 2 3 4 5 C 6 7 8 9 |
| Supplement 2: TLC chromatography of the supernatant of *S. marcescens* passing through the first column. S: Cell-free extract of prodigiosin by acetone. 1‒9: Fraction 1 to fraction 9 of cell-free extract of prodigiosin when passing through the silica gel column. |

| 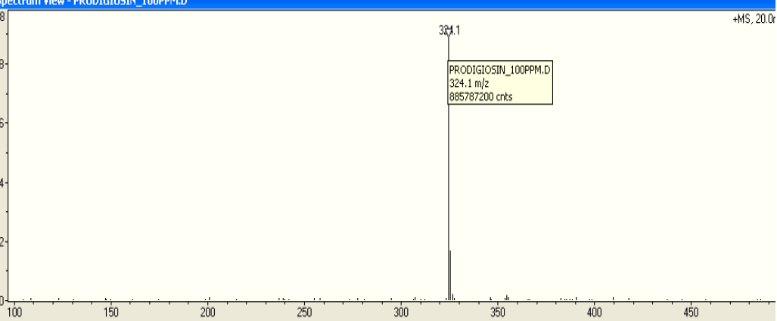 |
| --- |
| Supplement 3: MS spectrum of prodigiosin |
|  |

| 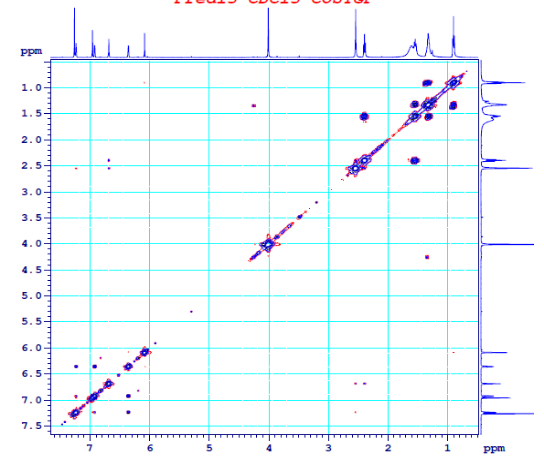 |
| --- |
| Supplement 4 COSY spectrum of prodigiosin |
|  |

| 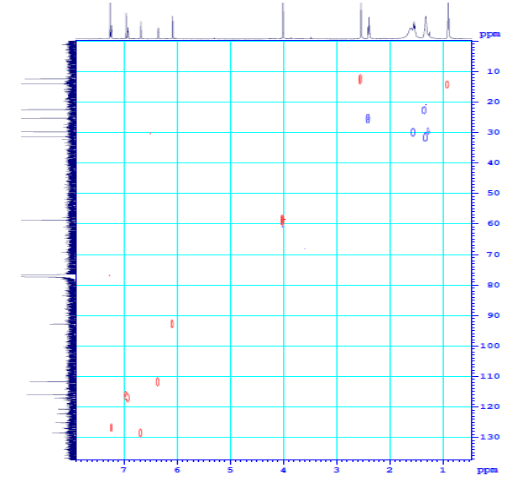 |
| --- |
| Supplement 5: HSQC spectrum of prodigiosin |

| 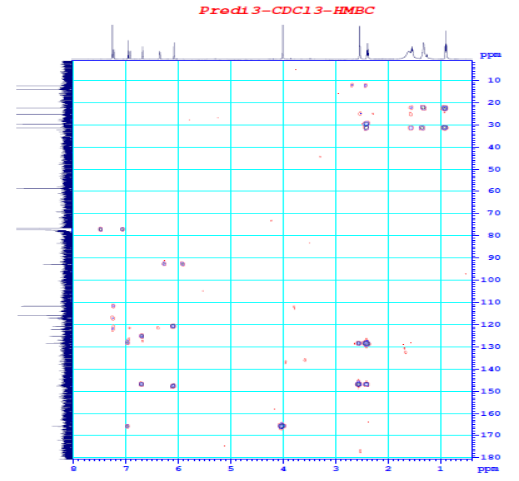 |
| --- |
| Supplement 6: HMBC spectrum of prodigiosin |
